# Assessment of Additively Manufactured Thermoplastic Composites for Ablative Thermal Protection Systems (TPSs)

**DOI:** 10.3390/polym17172338

**Published:** 2025-08-28

**Authors:** Teodor Adrian Badea, Lucia Raluca Maier, Alexa-Andreea Crisan

**Affiliations:** Composite Materials Laboratory for Aeronautical Field, Romanian Research & Development Institute for Gas Turbines—COMOTI, 220D Iuliu Maniu Av., 061126 Bucharest, Romania; teodor.badea@comoti.ro (T.A.B.); alexa.crisan@comoti.ro (A.-A.C.)

**Keywords:** TPS, oxy-acetylene torch, carbon fiber, glass fiber, 3D printing

## Abstract

This study focused on the thermal stability and ablative behavior assessment of five newly developed composite TPS configurations. All ten test samples were 3D printed via FDM using various fire-retardant thermoplastic materials, with and without reinforcement. Eight samples integrated a new thermal management internal air chamber conceptualized architecture. A prompt feasible approach for the flame resistance preliminary assessment of ablative TPS samples was developed, using an in-house oxy-acetylene torch test bench. Experimental OTB ablation tests involved exposing the front surface samples to direct flame at 1450 ± 50 °C at 100 mm distance. For each configuration, two samples were tested: one subjected to 30 s of flame exposure and the other to 60 s. During testing, internal temperatures were measured at two backside sample contact points. Recorded temperatures remained below 46 °C, significantly under the maximum allowable back face temperature of 180 °C set for TPSs. The highest mass losses were measured for PC and PETG FR materials, achieving around 19% (30 s) and, respectively, 36% (60 s), while the reinforced configurations showed overall only a third of this reduction. The study’s major outcomes were the internal air chamber concept validation and identifying two reinforced configurations as strong candidates for the further development of 3D-printed ablative TPSs.

## 1. Introduction

Thermal protection systems (TPSs) are essential for spacecraft re-entry, withstanding pressures >25 atm and combined convective/radiative heating that accelerates material degradation [1,2]. Ablative TPSs using fiber-reinforced polymers improve resistance via pyrolysis and char formation [3]. While legacy materials like Avcoat and PICA perform reliably, their scalability is limited by labor-intensive fabrication [4]. Recent advances include lyocell-based PICA [5], rayon-based felts [4], and natural rubber-modified CFPA, which improved fracture strain to 39.6% and reduced modulus while maintaining low density and ablation resistance [6]. Additive manufacturing (AM) has enabled TPS innovations, such as NASA’s DIW-printed cyanate ester (KREPE1, 2021) and phenolic dual-layer (KREPE2, 2023) systems, both validated by arcjet testing [7]. AM-processed SiC lattices with Qf/SiO_2_ aerogel reduced backside temperature by 112.73 °C, improved insulation by 13.85%, and tripled strength at 1000 °C for 1500 s [8]. According to [9], multi-scale hybridization via polymer additives, fiber modifications, and layered architectures significantly enhances the fire resistance and thermal stability of fiber-reinforced composite laminates (FRCLs), with numerical and experimental methods aiding performance prediction and design optimization. The review presents multi-scale hybridization strategies, such as nano-additive incorporation reducing the peak heat release rate by up to 43.9% and fiber hybridization enhancing post-fire strength by 240%, to significantly improve the fire resistance of composite laminates, supported by standardized fire testing and predictive modeling [9]. Further developments include CF/polysiloxane (UHTR) composites cured at 350 °C and benchmarked against MX4926 via TGA, MCC, OTB, and SEM [10], and CNT-aligned polysiloxane laminates (58 µm, 38 vol%) showing thermal stability but flame-induced delamination [11]. A VIP process for C-PICA (~0.29 g/cc) reduced waste vs. open molding, with minor voiding and delamination addressed in the scale-up [12]. Complementing these, a PEI (ULTEM™ 1010) nanocomposite (F9) with 20 wt% glass bubbles, 5 wt% nanoclay, and 10 wt% flame-retardant properties showed optimal FFF compatibility, a low heat flux, the lowest MCC heat release, and a high TGA char yield [13]. Despite F5 containing less nanoclay, 2.5 wt%, and exhibiting a higher peak surface temperature, the ~50 °C difference from F9 corresponds to only a 3% variation [13]. Legacy ablators like MCC-1 and P50 cork remain benchmarks under oxyacetylene testing, providing key data on recession, conductivity, and char for current TPS design [14]. As TPSs continue to evolve, innovative sandwich structures have gained attention for their low density and high performance. These designs incorporate various core configurations, such as lattice, honeycomb, corrugated, and bio-inspired forms, and use materials like laminated composites, ceramic matrix composites, and metals. Research has highlighted improvements in thermal resistance, mechanical strength, and manufacturability, making these sandwich structures a promising direction for next-generation TPSs [15].

Regarding material diversity and advancements, polymeric ablatives (PAs) prevail in TPS applications due to their tunable density, low cost, and high heat shock resistance, whereas nanostructured PAs (NPAs) enhance oxidation and ablation resistance in PICAs (<500 W/cm^2^) and nanoparticle-modified CCCs, though economical processing methods remain a limitation [16]. The development of ablative thermal protection systems for NASA missions has progressed from early materials, such as fiberglass–phenolic shingles with a peak heating of ~50 W/cm^2^ for mercury, to more advanced materials like Avcoat 5026-39G, which handled a peak heating of ~600 W/cm^2^ during the Apollo missions. Later, the Super-Lightweight Ablator (SLA-561V), with a density less than 50% of the Apollo ablator, was used for the Viking Landers, which experienced a peak heating of ~26 W/cm^2^ [17]. Carbon–phenolic and carbon–elastomeric ablative composites protect against extreme heat via endothermic processes, with performance influenced by filler type, fiber–matrix interaction, and mechanical properties; reported LAR and MAR reductions underscore the need for predictive modeling to optimize compositions and reduce development time, cost, and waste [18]. Carbon–carbon, carbon–phenolic, carbon–elastomeric, and carbon–ceramic ablatives, including UHTC-modified variants, exhibit thermal protection efficiency dependent on matrix chemistry, fiber architecture, and coating integrity; advancements in nano-reinforcement, densification control, and oxidation barriers highlight the necessity for optimized processing to balance performance, cost, and recyclability [19]. FRCLs improve fire resistance via multi-scale hybridization, including polymer toughening with micro/nano-particles, fiber hybridization, and multilayer laminates, supported by thermo-mechanical modeling and fire testing. Future work should integrate selective hybridization and numerical analysis (e.g., Abaqus) for optimized fire-safe composite design [9]. SiC-modified PAN-based carbon fiber phenolic composites (0–5 wt% SiC) showed optimal ablative and thermal performance at 3 wt% SiC, reducing linear and mass ablation rates by 28% and 13%, respectively, with improved strength and surface protection via SiO_2_ formation confirmed by XRD, SEM, and EDS. These findings highlight their potential for aerospace TPSs, warranting further filler optimization and testing under hypersonic conditions [20]. Also, TaC samples tested at ~2300 °C showed severe thermal shock cracking and oxidation below melting point, forming porous Ta_2_O_5_ layers and novel submicron grains, proving TaC unsuitable for ultra-high temperature (>2000 °C) applications despite its high melting point [21].

Pelin G. et al. [22] reported, in their work, the best results for a thermal protection system (TPS) based on a carbon fiber felt with a phenolic resin and 2% by weight nSiC, following Oxy-Butane Ablation Testing at 1300 °C. It recorded a backside temperature of 232 °C after 60 s exposure and 237 °C after 120 s, with a mass loss of 8.29% after 120 s. In their work, Elwan I. et al. [23] reported optimum results in a TPS composed of phenolic resin and 65% silica fiber. This material achieved a backside temperature of approximately 200 °C after 61 s and an erosion rate of 0.118 mm/s. Hao W. et al. [24] described the ablation tests results (performed at 1650 to 1697 °C, 100 W/cm^2^ flux, for 15 s, 30 s, 60 s, and 120 s) obtained on five different high-temperature thermoplastic materials 3D printed using FFF (e.g., PEI-ULTEM 9085/SABIC, PEEK1, PEEK2, PEKK, PEI-Modified ULTEM 1010). Four of the tested materials (except ULTEM 9085) were able to withstand 120 s exposure without disintegrating, while up to 60 s, they kept their original shape but experienced various amounts of intumescent or swelling behavior. For longer exposure times, the difference in residual mass becomes more apparent, among which PEKK consistently shows the highest residual mass, also exhibiting the highest char yield, while the two PEEK configurations exhibit the highest thermal decomposition temperature. Based on char yield and residual mass criteria, which are important parameters for ablation properties, PEKK is the overall most promising candidate amongst the five tested polymers.

Bench-scale fire testing provides a reliable alternative to full-scale TPS material evaluation. Oxy-acetylene torch testing remains a standard for ablative assessment [25], and simulations using Ansys Fluent closely match experimental heat flux and temperature data, confirming its accuracy for high-heat evaluation [26].

This research presents a significant advancement beyond the state of the art for ablative TPSs by proof of the thermal management capability (low conductivity and reduced amount of heat transferred by convection) of the new developed 3D-printed air chamber conceptualized structural architecture, also enhancing its lightweight feature.

## 2. Materials and Methods

### 2.1. Materials

**Figure 1:**
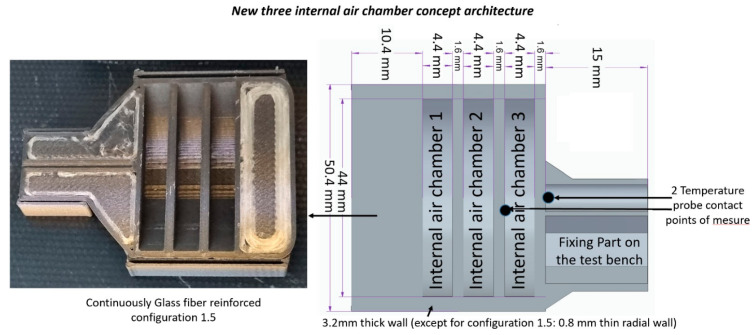
Sketch of the new developed ablative TPS configurations (integrating a new internal air chamber conceptualized architecture).

A total of five new developed 3D-printed ablative TPS configurations were developed via FDM technology. Two samples (one exposed to flame for 30 s and the other for 60 s) for each developed TPS configuration were tested in similar conditions. Different approaches (full infill and internal air chamber conceptualized architecture with/without reinforcement) were investigated. Various fire-retardant materials like Onyx FR-V0, PETG FR-V0, and PC FR-V0 with and without reinforcement were developed to integrate a new internal air chamber conceptualized architecture, except from the full infill Onyx FR-V0 (configuration 1.1). The original mass of the tested composite specimens ranged from 43 g to 45 g, depending on variables such as the type and morphology of reinforcement (short discontinuous or long continuous, carbon, or carbon and glass) and the FR reinforced materials, with the exception of fully infilled configuration 1.1, which weighed 61.5 g. The new three-internal-air-chamber conceptualized architecture proposed in the present study (revealed in Figure 1) relies on the basic principle of creating smaller airspaces, providing low thermal conductivity and reducing the amount of heat transferred by convection and radiation, in addition to being lightweight.

The thermal conductivity of air does increase slightly with increasing temperature, while it decreases with decreasing pressure. In the original concept, these air chambers have holes to communicate with the external medium, and when passing from air to vacuum, the thermal conductivity is effectively zero because there is no medium for heat transfer through conduction or convection, and it only occurs through radiation. Table 1 provides a summary of all configurations developed for the new ablative thermal shield protective systems. Onyx FR-V0 is a flame-retardant version of Onyx, which is nylon (polyamide 6) reinforced with 10% to 20% by volume of short carbon fibers according to [27] or 9% chopped fiber content in the matrix phase, as the image analysis of Sauer [28] revealed. Onyx FR achieved a V-0 rating on the UL94 flammability test while possessing similar mechanical properties to Onyx, having 1.2 g/cm3 density and a heat deflection temperature of 145 °C. Onyx has excellent properties, including its lightweight, mechanical properties, strong thermal stability, and resistance to ultra-violet (UV) radiation and chemicals. According to Hetrick et al. [29], the length of the carbon fibers in Onyx varies from 7.035 to 44.58 μm, and fibers are mainly oriented in the deposition direction of the material, potentially achieving strength comparable to 6061-T6 aluminum when additionally continuous carbon fiber-reinforced. Configuration 1.1 is a full infill configuration of Onyx FR-V0 with no concept integrated. Configuration 1.2.1 samples were 3D printed using Onyx FR-V0 with a three-internal-air-chamber architecture (a radially thick wall of 3.2 mm). Specifically, configuration 1.5. (consult Table 1) is 3D printed out of commercial high-strength high-temperature (HSHT) fiberglass material as continuous glass fiber 20.7%vol.-reinforced Onyx FR-V0, with a three-internal-air-chamber architecture and a thin radial wall configuration (0.8 mm—see Figure 1), while all the other geometrical features including the front wall (10.4 mm) and internal air chamber separation walls (1.6 mm) were kept the same as those for configuration 1.2.1, as shown in Figure 1. Additionally, two configurations (2 and 3, presented in Table 1) were developed using flame-retardant versions of Polyethylene terephthalate glycol (PETG-FR V0) and polycarbonate (PC-FR V0), also integrating the three-internal-air-chamber architecture (a radially thick wall of 3.2 mm), while all the other geometrical features such as the front wall (10.4 mm) and internal air chamber separation walls (1.6 mm) were kept the same as those for configuration 1.2.1, as illustrated in Figure 1. PETG-FR V0 is a halogen-free, flame-resistant 3D printing material, the filament being designed according to UL 94 V0* flammability rating requirements, and provides low shrinkage and relatively high tensile strength, with 1.2 g/cm^3^ density. PC FR-V0 is a fire-retardant polycarbonate filament displaying strength and toughness whilst also achieving a V0 score in the UL94 flame retardancy test, with 1.26 g/cm^3^ density. For space applications, where high reliability and safety are critical, a V0 rating is often preferred for components exposed to potential ignition sources. However, while UL 94 V0 is a good baseline, other factors like outgassing, thermal stability, and radiation resistance also need to be considered when choosing materials for space environments. All ten developed composite ablative TPS additively manufactured configurations/samples are listed below in Table 1.

### 2.2. Additive Manufacturing Technology

Ablative TPS test samples were manufactured using additive manufacturing Fused Deposition Modeling (FDM) technology. The tested samples were 3D printed using a Prusa XL 3D printer (Prusa Research a.s., Prague, Czech Republic) (a 1.75 mm filament diameter for all Onyx FR-V0, PETG-FR V0, and PC-FR V0 materials), while continuous glass-reinforced Onyx FR-V0 configuration 1.5 was developed on a Markforged X7 3D printer (Markforged Holding Corporation, Waltham, Massachusetts, USA) (310 µm fiber filament diameters). The nozzle temperature for the continuous reinforced Onyx FR-V0 configuration was set to 275 °C, whereas the printing bed was non-heated, the layer height was preset at 100 µm and one shell was used. The nozzle diameter of 0.4 mm and 100% infill were similar on both 3D printers. The printing temperatures for samples manufactured using the Prusa 3D printer were 110 °C bed temperature and 290 °C nozzle temperature (Onyx FR-V0 samples), 105 °C bed temperature and 270 °C nozzle temperature (for PC-FR V0 samples), and 80 °C bed temperature and 230 °C nozzle temperature (PETG-FR V0 samples), and the layer heights were 0.2 mm, the infill was rectilinear and two shell walls were used.

### 2.3. Fiber Reinforcement Considerations

Carbon fibers have excellent thermal stability and can withstand very high temperatures (often above 3000 °C in inert atmospheres). Nevertheless, when exposed to high heat, in the presence of oxygen, carbon fibers can oxidize (burn), typically starting around 600 °C, and oxidation leads to degradation, structure breakdown and the loss of mechanical strength. In the present study, excluding configurations 2 and 3, all samples were 10% vol. short carbon fiber-reinforced. Moreover, additional continuous glass fiber-reinforced configuration 1.5 was developed to evaluate the contribution of the additional continuous reinforcement phase to the thermal resistance and ablation performance of the newly developed 3D-printed TPS configurations beyond the influence of the novel internal air chamber architectural concept. Although glass fibers start to soften at high temperatures typically around 800 °C, they do not readily ignite or burn and are considered non-combustible. The main component of glass, silica, is already in its highest oxidation state and thus does not react with oxygen, meaning it will not burn in a fire. The matrixes embedding the fibers in different configurations (short carbon/short carbon and long continuous glass fiber), although of a thermoplastic nature, are all modified FR blends. When exposed to very high temperatures, they are the first that undergo thermal degradation, melting, vaporization or sublimation, followed by the fibers. However, the abovementioned information must be deeper analyzed since the role of fiber reinforcement in the composite burning process is significantly complex, depending on the fiber type, its quantity, orientation, length, thermal conductivity, and size agent.

### 2.4. Oxyacetylene Flame Test Bench

**Figure 2:**
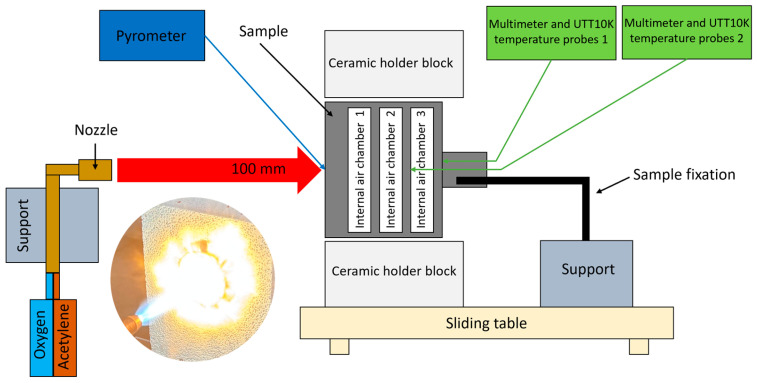
Oxyacetylene torch test bench set up (OTB ablation testing).

A custom-designed oxyacetylene torch test rig was developed internally to perform the high-temperature oxidation test. The schematic of the torch test facility OTB for ablation testing is illustrated in Figure 2.

Two oxygen and acetylene reservoirs with flow meters and pressure gauges, along with a torch as the heat source, were used. Oxygen and acetylene were fed through a welding nozzle (LS14 model inner No. 4 nozzle, the RHONA GCE group, Steinhausen (Zug), Switzerland) to produce a high-temperature (1450 ± 50 °C) oxidizing flame. For testing, the samples (the 50 mm diameter samples, with geometry features given in Figure 2) were positioned in a ceramic holder block with thermal resistance up to 1450 °C and relatively low thermal conductivity (0.33 W/m·K). The holder integrated a 51 mm diameter circular hole for sample accommodation. The samples were mechanically fixed in a rigid hold-up attached to a mobile roller plate equipped with a locking point system. Thus, the samples were mounted in the same exact position into the holder and exposed to the flame by slowly manually advancing the mobile roller plate to the same target point (to enable ease in the alignment and position the direct flame into the center of the sample frontal wall). The distance between the nozzle and the sample was 100 mm to achieve the desired peak temperature and heating profile. Each developed configuration was tested using two samples: one exposed to a flame for 30 s and the other for 60 s. Then, the sample was retreated by pulling the mobile roller plate out of the flame field to extract the tested sample, allowing it to cool down naturally, and then we mounted the next test sample. In order to obtain quantitative and repeatable tests, this set up ensured that the flame remained continuously lit from the beginning of the tests until the end of the campaign. During testing, the peak temperature at the center of the sample’s front surface was recorded using a infrared pyrometer (Sonel DIT-500 from SONEL S.A., Świdnica, Poland). The back face internal temperatures were recorded at two contact points located on the rear walls of the second and third air chambers, as viewed from the front of the sample (as shown in Figure 2), using UTT10K UNI-T temperature measure probes with a range up to 260 °C and a high accuracy of ± 0.75%, both connected to two digital multimeters (UT131C from UNI-T (UNI-Trend Technology Co., Ltd., Shenzhen, China) and, respectively, SMA 19 from Somogyi Elektronic Kft., Győr, Hungary).

### 2.5. Post-Test Analysis

Visual inspection and morphology analysis, along with weight and thickness measurements, were performed following the OTB ablation tests. Weight measurements were performed using a Kern PLJ 510-3M (KERN & SOHN GmbH, Balingen, Germania), analytical balance providing ±0.001 g precision. Thickness measurements were made using a Mitutoyo CD-P15P (Mitutoyo Corporation, Kawasaki, Japan), digital vernier caliper model with a measuring range of 0–150 mm and a measuring accuracy of 0.01 mm.

## 3. Results

### 3.1. Morphological Analyses

**Figure 3:**
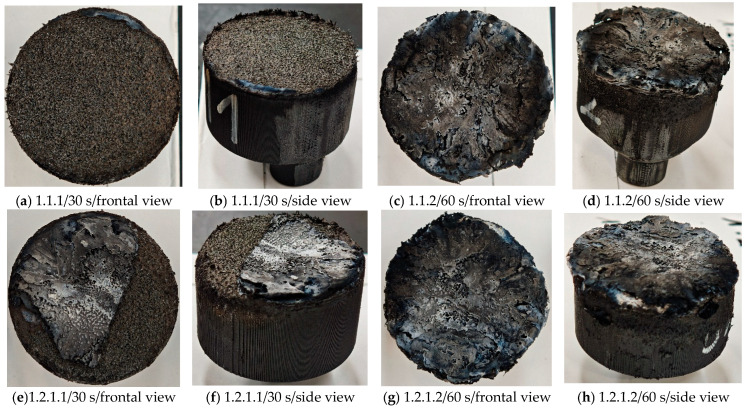

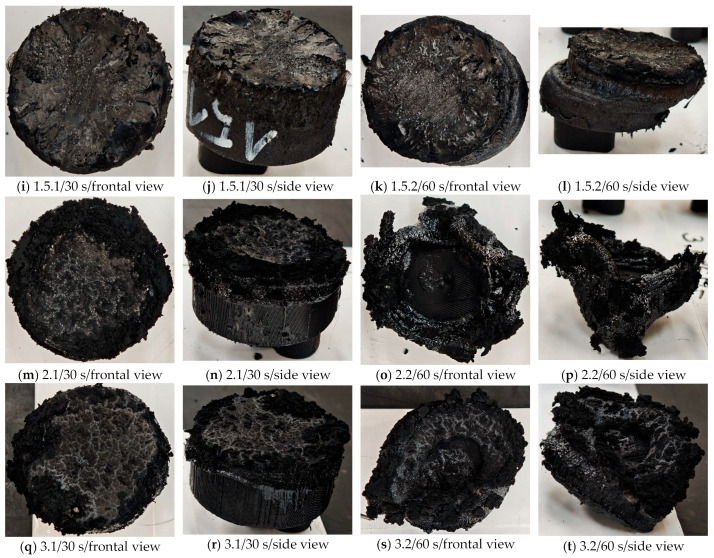
Images of the samples after OTB ablation testing: configuration/exposure time/frontal view/side view post test prior to char removal.

Images of the samples after OTB ablation testing are presented in Figure 3. As a general observation, the sample front faces are all covered with a carbon-rich char layer with dark-brown regions, indicating that the expected oxidation occurred. Unlike traditional ablatives that form a thick char layer, thermoplastic ablatives often ablate with high rates of gas formation at lower surface temperatures, and the char layer is thinner. 

Nevertheless, as it can be seen, the char layer acts as a barrier to heat transfer; its porous structure provides insulation and potentially can also re-radiate some of the absorbed heat, and as the surface of the char layer erodes away through ablation, it carries away heat energy, further protecting the underlying material. The structural materials Onyx FR V0, PETG FR V0, and PC FR V0 under study are halogen-free fire-retardant materials, achieving by a combination of additives their fire-resistant properties through both gas-phase and condensed-phase mechanisms. The first involves the release of non-flammable gases or the capture of free radicals during combustion, which inhibit the flame, while the last promotes the formation a char layer on the surface of the material during combustion, this acting as a shield, insulating the underlying material and reducing the rate of heat release. These mechanisms worked synergistically to reduce heat release, smoke production and the spread of flames. Likewise, these materials exhibit close values of the Limiting Oxygen Index (LOI) parameter, typically above 30%, thus requiring a high concentration of oxygen to sustain combustion, making it suitable for applications demanding fire resistance. Although a carbon-rich char layer was observed covering the tested samples, some depleted char regions were observed on some samples due to char detachment during the test and post-test sample extraction, whereas there was some adhesion for the other samples, possibly due to the formation of liquid phases at the test temperature and their subsequent solidification on cooling. Morphological observations revealed that in the case of Onyx FR V0 degradation, the formation of carbonyl groups due to reactions with oxygen at an elevated temperature led to the formation of a more stable and protective char layer compared with the PETG FR V0 and PC FR V0 configurations.

Furthermore, the fire exposure behavior of the new developed configurations was observed to be significantly different between the tested configurations. Referring to the 30 s exposure time samples, it is evident that configuration 2 and 3 samples behaved differently in similar conditions compared to the other tested configurations. This lower thermal resistance behavior that lead to the melting, dripping and complete disintegration of the overall structure for both mentioned configurations became more explicit after 60 s exposure time, while after 30 s, only a partial consumption of the frontal wall materials was observed for both configurations (Figure 3). This behavior can be attributed potentially but not exclusively to the reinforcement phase, which is not present in configurations 2 and 3, since in terms of bulk material thermal conductivities, no major disparities were observed (being around 0.2 W/m·K). The 10% by volume of short carbon fibers (configurations 1.1 and 1.2.1) and additional continuous glass fiber frontal reinforcement (configuration 1.5) not only provide high strength and stiffness but also, by their morphology and distribution, lead to anisotropic thermal conductivity (0.9 W/m-K in the flow direction and 0.3 W/m-K perpendicular to the flow direction) [30] in these reinforced materials. This anisotropy played an important role to quickly dissipate heat away from the ablation surface in one direction (e.g., along fibers) while restricting heat flow in other directions (e.g., into the material). This helped to prevent excessive heat buildup at the surface and reduced damage. Indeed, the burning mechanisms were different for bulk thermoplastic and composite configurations due to the fiber–matrix interaction and heat transfer. The interface region between the matrix and reinforcement in composite materials played a crucial role in their burning behavior. Reinforcing fibers in the composite influenced the burning behavior by conducting heat differentially depending on their direction, affecting the temperature distribution within the composite. In the first stage, the melting and dripping of the thermoplastic material took place, and the carbon-rich char layer covering the tested samples and acting as a barrier to heat transfer was locally removed by flame abrasion in the central region of the front sample wall, revealing the matrix–reinforcement interface (specifically for configuration 1.5). The surface morphology analysis showed that while the chars of both configurations 2 and 3 were quite porous and fragile, detaching after 60 s exposure and leading to the structural failure of the samples, the char layers did not easily detach from the remaining virgin material on the other reinforced configurations. Furthermore, recession rate, thickness, and mass loss analyses were performed following char layer elimination by brushing the surface.

### 3.2. Recession Rate

The recession rate, referring to the rate at which the material is consumed or eroded due to ablation, was calculated by dividing the thickness loss of the specimen by the exposure time (the difference between the original thickness and the final post-burning thickness). Like Allcorn et al. in [31], for this measurement, the thickness after testing is considered to be the thickness of the post-tested virgin material, meaning that the recession value is a measure of the progression of the reaction layer into the virgin material and does not factor in the thickness of the char on the surface. Recession is an indicator of the time duration of thermal protection, so low values are desirable. Table 2 summarizes the results of recession rates and provides cross-section views of all post-tested samples.

All tested sample surface images, along with section views and post-burning thickness measurements (of the frontal wall before the first air chamber, as shown in Table 2) following char layer elimination by brushing, were analyzed. It is important to stress that the second and third walls separating the air chambers were not affected, keeping their dimensions and structural integrity for all samples exposed for 30 s. By increasing time exposure to 60 s, it can be observed that the Onyx FR V0 reinforced configurations kept their structural integrity, except for configuration 1.5 where the frontal wall collapsed due to the thin radial walls and lack of reinforcement in this specific region. With respect to the recession rate, it can be observed that configurations 1.2.1 and 1.5 display lower values, indicating an increase in thermal resistance, compared to the other tested configurations. Configuration 1.5 exhibits a lower recession rate, potentially attributed to both carbon and glass reinforcements: short/chopped carbon fiber that ensures anisotropic thermal conductivity quickly dissipates heat away from the ablation surface in the fiber direction while restricting heat flow into the bulk material, whereas continuous glass fiber reinforcing the front wall of the sample provides an additional thermal and abrasion barrier for the material. Configuration 1.2.1 compared to configuration 1.5 shows a slightly higher recession rate (due to the lack of additional glass reinforcement); nevertheless, it shows improved thermal resistance compared with bulk full infill configuration 1.1, and this is likely due to the new conceptualized architecture developed within the present study, which ensures lower thermal conductivity due to the smaller airspaces created within the material structure. By contrast, configurations 2 and 3 showed a significant increase in recession rates. This is not only due to the lack of local reinforcement but to different heat transfer through PC FR-V0 and PETG FR-V0 materials. This different heat transfer is linked to the specific heat capacity intrinsic parameter that significantly impacts a material’s ablation resistance. Onyx FR V0 exhibits a room-temperature high specific heat capacity of 2050 J/(kg K) [27] and can absorb more heat energy before its temperature rises, leading to better resistance against ablation. On the other hand, PETG FR V0 (1200 J/kg·K) and PC FR V0 (1170 J/kg·K) materials have similar specific heat capacities, indicating lower ablation resistance.

**Figure 4:**
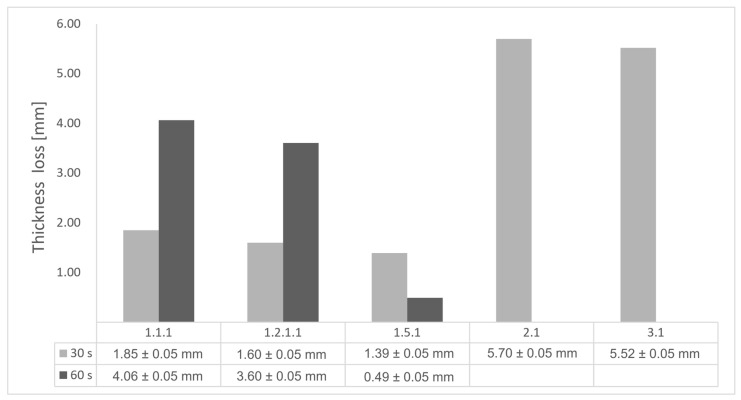
Thickness loss chart of tested configurations following char layer elimination.

All the above observations are consistent with the thickness losses reported in Figure 4. The heat conduction in the through-thickness direction was progressive for all tested samples, transferring the thermal energy through the structural material via molecular collisions, gradually, layer by layer, from the hotter to the colder side. In the case of configurations 2 and 3, there was a nearly homogenous transfer, while in the case of reinforced configurations, thermal conductivity anisotropy brought about by the chopped/short carbon fiber reinforcement dissipated heat away from the ablation surface in one direction (e.g., along fibers) while restricting heat flow in other directions (e.g., into the material). Furthermore, configuration 1.5 revealed the formation of delamination cracks and pores between the glass front plies and the bulk carbon-reinforced Onyx FR V0 material. Their formation is caused by the reactive volatiles in the decomposition region and evaporated water near the rear-face region of the composite flowing through the char layer towards the hot surface. During the process, some of them can be trapped due to the low gas permeability of the composite, leading to higher internal pressure and the expansion of the composite.

The thickness losses indicate clearly that Onyx FR V0 reinforced configurations showed only one-third thickness loss compared to configurations 2 and 3. Likewise, following 30 s time exposure, a comparison between configurations 1.2.1 and 1.5 indicates that the additional glass fiber continuous reinforcement in the frontal wall thickness lead to a lower loss of the thickness.

Glass fiber reinforcement layers not only contribute to higher structural stiffness but, through their very low thermal conductivity, act like frontal barrier thermal protection. Nevertheless, the decrease in wall thickness (thin wall thickness 0.8 mm for configuration 1.5) compared to configuration 1.2.1 (thick wall thickness 3.2 mm), resulted in a catastrophic destruction of the sidewall (radial) when increasing the exposure time to 60 s. Thus, a clear path for the optimization of configuration 1.5 is to increase the wall thickness to 3.2 mm as conducted for configuration 1.2.1 and additionally increase the glass fiber continuous reinforced region in order to maintain glass fiber benefits even after longer exposure times.

### 3.3. Mass Loss Assessment

**Figure 5:**
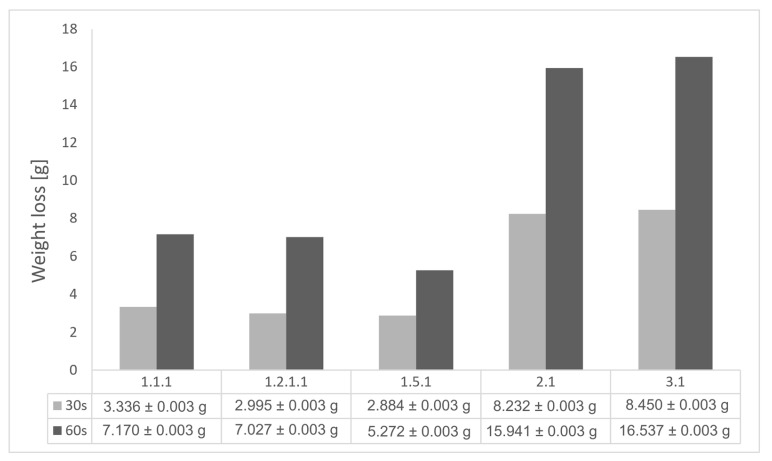
Mass loss results for OTB ablation testing of new composite TPS configurations.

Mass losses were assessed after 30 s and 60 s exposure time, following char layer elimination by brushing. The results of the mass loss measurements for the all tested configurations are indicated in Figure 5, and follows roughly the same trends as the results for recession rates and thickness loss. The best performing weight loss values match up to those for minimal mass loss for configurations integrating the new air chamber thermal management concept. The weight percentage losses vary from 6 to 11.5% for the glass/carbon-reinforced configuration 1.5 and, respectively, 6 to 16% for carbon-reinforced configuration 1.2.1 when increasing the time exposure from 30 s to 60 s.

Mass loss values can provide an indication of the time duration that ablatives can offer effective thermal protection. Configuration 1.1 (full infill) showed, as expected, low mass loss; however, unexpectedly, the lower mass loss value was measured for configuration 1.5 and, respectively, configuration 1.2.1, indicating that the new air chamber conceptualized architecture provided low thermal conductivity and reduced the amount of heat transferred by convection and radiation, creating these smaller airspaces within the material’s structure. Low mass loss values, under 7% mass reduction, were obtained for Onyx FR V0 reinforced configurations, whereas for configurations 2 and 3, the mass loss was nearly 19% after 30 s exposure time. This indicates again the benefits of reinforcement and change in local mechanisms of heat transfer within the thermoplastic composites. Although configurations 2 and 3 showed nearly similar behavior for both exposure times, mass loss was measured to be slightly higher for configuration 2 (PC FR V0). This similar trend in mass loss was observed for the higher exposure time of 60 s, where low mass loss values, under 16%, were obtained for Onyx FR V0 reinforced configurations, whereas for configurations 2 and 3, mass loss achieved nearly around 36%. As discussed above, although configuration 1.5 registered a radial sidewall failure due to its decreased thickness (wall thickness 0.8 mm compared to configuration 1.1—wall thickness 3.2 mm), leading to front wall sliding during dripping, it showed lower mass loss when exposed to both 30 s and 60 s. Consequently, since configuration 1.5 displayed typically better thermal resistance, these results can be considered quite promising for further development. A clear path for the optimization of configuration 1.5 is to increase the wall thickness to 3.2 mm as conducted for configuration 1.2.1 and additionally increase the glass fiber continuous reinforcement in both frontal and radial walls in order to maintain glass fiber benefits even after longer exposure times.

### 3.4. Temperature and Time Analysis

**Figure 6:**
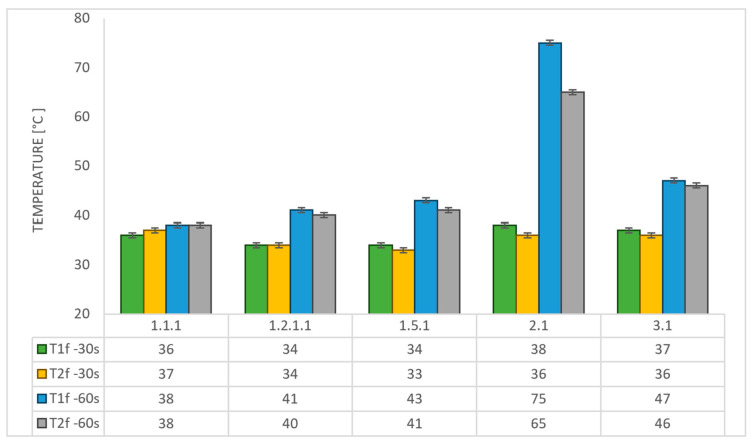
Temperature recorded upon exposure time for OTB ablation tested configurations.

The two embedded temperature measuring probes connected to two digital multimeters in each sample recorded the temperature from the beginning until the end of the test for both 30 s and 60 s time exposures. Figure 6 below provides the final temperature values registered for each sample for both time exposures. A general remarque stands out with respect to the measured temperatures at both locations: contact points located on the rear walls of the second and third air chambers, as viewed from the front of the sample (as shown in Figure 6).

With the exception of configuration 2 (after 60 s of exposure), none of the tested configurations caused the recorded temperature to rise above 46 °C—comfortably below the maximum allowable limit of 180 °C for the TPS back face temperature—thus ensuring the spacecraft and its components remain within design integrity. Configurations 1.5 and 1.2.1 and, although with a different temperature increase rate, configuration 3 exhibit comparable behavior, indicating an effectiveness in providing thermal protection by the integration of the new air chamber concept in a synergistic effect with the reinforcement phase in the case of first two mentioned configurations. The increase in temperature ranged from 5 to 10 °C, for both measuring locations, when increasing time exposure from 30 s to 60 s. Although the temperature recorded indicates configuration 1.1 as an optimum choice, it cannot be an option for further optimization due to its high mass compared to configurations integrating the new thermal concept.

## 4. Short Discussion

Although the main output of the present research relies on the thermal management through the new internal air chamber conceptualized architecture and the integration of 3D printing technology as a valuable tool to obtain complex architectures, with low resource use in terms of time and costs, the structural materials used to additively manufacture the samples also remain a key factor. It was clearly evidenced that the reinforcement phase within the FR structural material plays a crucial role in terms of the heat transfer mechanisms and structural integrity of the samples during OTB ablation testing. Furthermore, when designing the radial or separation walls’ thickness between the air channels and the frontal wall thickness, it is also instructive to consider the anisotropic thermal conductivity of a fiber-filled material, recalling that thermal conductivity is high in the fiber-aligned direction. This features will be under analysis when also applying continuous fiber reinforcement by means of Continuous Filament Fabrication (CFF) technology as part of he FDM process. Therefore, since configuration 1.5 displayed notable thermal resistance, these results can be considered quite promising for further development. As mentioned above, an effective optimization strategy for configuration 1.5 involves increasing the wall thickness to 3.2 mm, as implemented in configuration 1.2.1, and enhancing the continuous glass fiber reinforcement in both the frontal and radial walls to preserve the benefits of glass fiber even under prolonged exposure. In addition, our future research study intends to address other types and new architectures of reinforcement along with potential surface coatings to further increase thermal resistance and ablation.

Comparing our results with the work of Pelin G. et al. [22], we observed a significantly lower backside temperature, which rose to a maximum of 50 °C. The mass losses were comparable, ranging from 6% to 11.5% for the glass/carbon-reinforced configuration, after a maximum exposure time of 60 s. Although the materials analyzed (thermoset vs. thermoplastic) were different, both TPSs demonstrated improved behavior compared to the cork-based ablative ones investigated in the study.

Furthermore, when comparing our results with those of Elwan I. et al. [23], we found a significantly lower backside temperature (maximum 50 °C) and lower or comparable erosion rates after a maximum 60 s exposure, depending on the specific TPS configuration. When contrasting our findings with the work of Hao W. et al. [24], we noted that our backside temperature of 50 °C after 60 s was considerably lower than the 370 °C reported for a similar exposure time. Additionally, the reported residual mass for PEKK without char (after 60 s) was 66%, which is lower than the 87% residual mass value obtained for the optimum configuration in our current study.

## 5. Conclusions and Further Outlook

Two newly developed ablative TPS configurations featuring an internal air chamber concept, produced via additive manufacturing, showed promising thermal stability and ablative characteristics results. Three-dimensional-printed composite TPS material enabled complex geometries and structural architecture development, with low resources in terms of time and costs, offering the possibility of in space in situ manufacturing. Various fire-retardant structural materials including Onyx FR-V0, PET-G FR-V0, and PC-FR-V0 were investigated, ranking the first one as a promising choice. Based on the results of this study, glass/carbon-reinforced configuration 1.5 outperformed the baseline (full infill configuration 1.1) in terms of the recession rate and exhibited comparable mass loss results following OTB ablation testing while showing lower mass loss when exposed to both 30 s and 60 s compared to all configurations tested. Another noteworthy configuration, the 1.2.1 concept at baseline, showed a slightly higher recession rate and mass loss compared to glass/carbon reinforced configuration 1.5, but it kept its structural integrity after 30 s and 60 s exposure during OTB ablation testing due its thicker radial wall. Through this study, it was proven that the novel internal air chamber design is a viable candidate for additional analysis aimed at improving thermal management in TPS ablative systems. Consequently, future research will focus on optimizing geometric features and structural architectures. In particular, enhancing configuration 1.5 entails raising the wall thickness and expanding the continuous glass fiber-reinforced region to preserve its performance benefits over prolonged exposure. Furthermore, building on the advancements in additive manufacturing (AM) for TPSs, this work leverages the capabilities of 3D printing to fabricate complex geometries with tailored thermal and mechanical properties.

## 6. Patents

A national patent request was filled prior to the present work in relation with the new internal air chamber concept *Heat shield obtained through additive manufacturing with low weight reference and air/vacuum cushions intended for space applications*, Teodor-Adrian Badea, Alexa Crisan, Raluca Maier, reference no. A/00073- OSIM: 26.02.2025

## Figures and Tables

**Table 1:** The specifications and nomenclature of the as-produced new composite ablative TPS additively manufactured configurations/samples (integrating a new internal air chamber conceptualized architecture) ^1^.

Material	Configuration Code ^1^	Sample Code/Mass *			
		Sample (30 s)	Mass [g] *	Sample (60 s)	Mass [g] *
Onyx FR-V0	FR/R/Full infill	1.1.1	61.45	1.1.2	62.4
	FR/R/TW/3 AC	1.2.1.1	43.98	1.2.1.2	44.97
	FR/R/CGF-F/TnW/3 AC	1.5.1	45.44	1.5.2	45.68
PC FR-V0	FR/NR/TW/3 AC	2.1	43.22	2.2	43.26
PETG FR-V0	FR/NR/TW/3 AC	3.1	45.21	3.2	45.8

^1^ FR—fire-resistant; R—reinforced (Onyx FR-V0 reinforced with 10% chopped carbon fiber); NR—not reinforced; TW —thick radial wall of 3.2 mm; TnW—thin radial wall of 0.8 mm; AC—air chamber; CGF-F—continuous glass fiber reinforced only frontal wall area; *—mass weight were determined prior to tests (excluding fixation parts).

**Table 2:** Recession rates, surface and cross-section view of exposure time/frontal tested configurations.

30 s/Total Erosion (mm)		Erosion Rate (mm/s)	60 s/Total Erosion (mm)		Erosion Rate (mm/s)
1.1.1		0.062 ± 0.002 mm/s	1.1.2		0.068 ± 0.002 mm/s
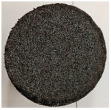	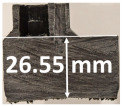		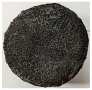	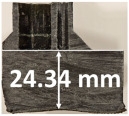	
1.2.1.1		0.053 ± 0.002 mm/s	1.2.1.2		0.06 ± 0.002 mm/s
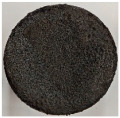	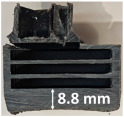		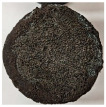	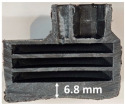	
1.5.1		0.046 ± 0.002 mm/s	1.5.2		0.008 ± 0.002 mm/s
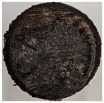	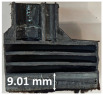		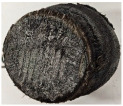	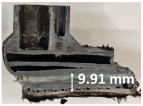	
2.1		0.19 ± 0.002 mm/s	2.2		NA
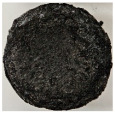	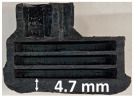		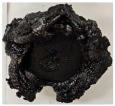	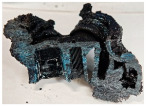	
3.1		0.184 ± 0.002 mm/s	3.2		NA
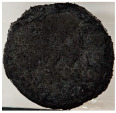	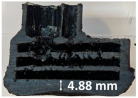		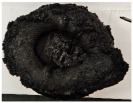	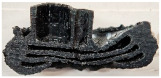	

## Data Availability

No new data were created or analyzed in this study. Data sharing is not applicable to this article.
